# Case report: New perspectives on gait initiation strategies from a case of full toes amputation in a professional mountain climber

**DOI:** 10.3389/fnhum.2024.1463249

**Published:** 2024-09-26

**Authors:** Jorge L. Storniolo, Veronica Farinelli, Mattia Onesti, Luca Correale, Leonardo A. Peyré-Tartaruga, Roberto Esposti, Paolo Cavallari

**Affiliations:** ^1^Human Physiology Section of the Department of Pathophysiology and Transplantation, Università degli Studi di Milano, Milano, Italy; ^2^Human Locomotion Laboratory (LOCOLAB), Department of Public Health, Experimental Medicine and Forensic Sciences, University of Pavia, Pavia, Italy; ^3^LaBiodin Biodynamics Laboratory, School of Physical Education, Physiotherapy and Dance, Universidade Federal do Rio Grande do Sul, Porto Alegre, Brazil; ^4^Laboratorio Sperimentale di Fisiopatologia Neuromotoria, IRCCS Istituto Auxologico Italiano, Meda, Italy

**Keywords:** bilateral toes amputation, human, anticipatory postural adjustments, posturography, gait analysis

## Abstract

**Introduction:**

We studied the postural behaviour of a 52-year-old professional mountain climber who underwent bilateral amputation of all five toes after severe frostbite.

**Methods:**

Two tasks were examined: static posturography (SP) and gait initiation (GI), both performed barefoot and with prosthetic shoes. During SP, the participant kept the upright stance for 30 s while an optoelectronic system with reflective markers recorded feet position and body sway, and two force plates measured the Center of Pressure (CoP) displacement and Ground Reaction Force (GRF) of each foot. During GI, the participant stood on the force plates for at least 10 s and then spontaneously started walking, while optoelectronic system was used to monitor heel-off events; wireless EMG probes recorded the anticipatory postural adjustments (APAs) in trunk and lower limb muscles.

**Results:**

Compared to shod condition, during barefoot SP the participant showed a reduced anteroposterior (AP) and mediolateral (ML) extension of the Base of Support (BoS), and the whole-body CoP shifted about 7 mm more anteriorly, approaching the “safer” geometric center of the BoS. Despite this difference, the AP and ML ranges of CoP oscillations were similar in both conditions. In GI, the trunk dorsal muscles showed different APA patterns: when barefoot they were excitatory in the trailing and inhibitory in the leading side while they were bilaterally inhibitory when shod.

**Discussion:**

In parallel to CoP shift toward a “safer” position in SP, in barefoot GI the body rotation toward the trailing side may reveal a more “cautious” approach; this also shows that different postural strategies may be adopted in GI by one and the same individual.

## 1 Introduction

Any amputation presents various challenges in performing daily activities and when it affects the lower limbs the postural control may be greatly impaired. Postural control is classically considered in a two-fold framework: static and dynamic balance.

Static balance derives from the capability of the postural control system to keep the vertical projection of the body center of mass (CoM) within the feet contact area, ensuring postural stability in upright standing. Body oscillations are quantitatively evaluated by baropodometry (pressure plates) or by Static Posturography (SP) using force plates (Fullin et al., 2022). In particular, the toes are crucial for providing stability during quiet standing or simply in shifting body weight from one foot to another (Chou et al., 2009). Even incomplete toes amputation may result in difficulties in evenly distributing body weight between the feet, as well as in controlling the position of the center of mass (Forczek-Karkosz et al., 2021). This imbalance may lead to compensatory movements of other body parts; moreover, static balance could be compromised due to the reduced proprioceptive feedback from the forefoot (Felicetti et al., 2021).

Dynamic balance refers to maintaining stability during voluntary movement and locomotion. For gait assessment the gold standard instrumental method is the 3D Motion Analysis; in particular the optoelectronic system is used for the whole-body kinematic assessment (De Blasiis et al., 2022) and wireless surface EMG to evaluate timing of muscles activation (Campanini et al., 2022). For individuals with missing toes, such tasks may become more challenging, indeed proper push-off during walking or running might be affected by the reduced lever-arm between the ankle and the foremost point of contact between the foot and the ground. Such deficits have been illustrated in walking and running by Chou et al. (2009). Aside from toe amputation, other forefoot issues such as rheumatoid arthritis, diabetes, and hallux valgus may lead to decreased walking speed, due to reduced spatiotemporal parameters, also leading to gait instability (Nix et al., 2013; Sacco et al., 2014). In this framework, it is interesting to note that no data is available in literature regarding the Gait Initiation (GI) task in toes amputees. This seemingly easy task indeed poses a great challenge for dynamic balance, as it requires to develop a controlled “forward fall” of the body. This is accomplished by a coordinated pattern of pre-programmed postural actions (Anticipatory Postural Adjustments, APA) in trunk and leg muscles that accompany the recruitment of ankle prime movers (cfr. Farinelli et al., 2021).

In this case report, we analyzed GI as well as SP in a professional mountain climber who underwent bilateral amputation of all toes in 2008 and, after one year of rehabilitation and by wearing prosthetic shoes, regained the ability to stand in upright position, to walk and even to climb mountains. The analyses were performed in both barefoot condition and while wearing the prosthetic shoes, so as to i) quantify the residual deficits in static and dynamic balance when barefoot, ii) assess the efficacy of the shoes and iii) test whether the APA pattern in GI changes in the barefoot *vs.* shod conditions, maybe revealing different strategies in accomplishing such a task.

Furthering the comprehension of how amputation affects static and dynamic balance would be crucial for healthcare professionals. By identifying potential issues related to postural control, appropriate interventions like prosthesis adjustment or specialized training programs may be implemented more effectively.

## 2 Materials and methods

### 2.1 The participant

**FIGURE 1:**
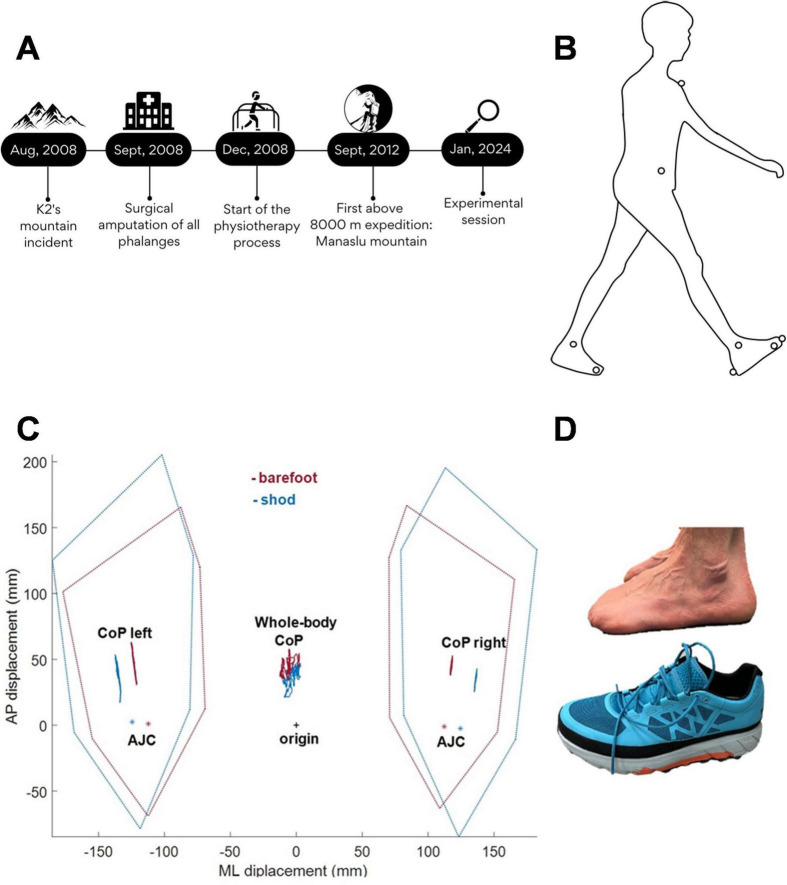
**(A)** Timeline of relevant data from the episode of care. **(B)** Side view of marker placement: heel, lateral malleolus, head of fifth metatarsal and stump of the big toe, as well as ASIS and acromion, are illustrated on the right body side; medial malleolus and head of first metatarsal are illustrated on the left foot. **(C)** Representative trial with CoPs displacement during static posturography in barefoot (red) and shod (blue) conditions, as depicted in the inset of panel **(D)**. The black cross marks the origin i.e., the midpoint of the ankle joint centers (AJC, asterisk); the dotted lines represent the base of support for each foot.

The participant was a 52-year-old professional mountain climber (male; height: 1.79 m; weight: 82 kg) who took part in an expedition to reach the top of K2, the second highest mountain in the world. Several accidents generated an extremely difficult situation and, due to severe frostbite, all his left and right toes were amputated, leaving him with a foot length of 23 cm. A year later, he began posture and walking recovery with the aim of reaching other 8000 m mountain peaks, a result attained more than 10 years ago (Figures 1A, D). The Ethical Committee “Comitato Etico di Ateneo dell’Università degli Studi di Milano” approved the study procedure (counsel 23/23). The subject gave written informed consent in compliance with the Declaration of Helsinki.

### 2.2 Experimental setup

The experimental session included Static Posturography (SP) and Gait Initiation (GI) tasks. The room’s temperature and lighting were controlled to ensure optimal conditions for the participant’s trials. For the SP task, the participant stood quietly with eyes open, in a comfortable position, placing his feet on two separate force plates. He was instructed to look straight ahead (targeting a chair 3 meters away) with his arms along his sides, remaining in upright static condition for 30 s. For GI, instead, the participant was asked to stand on the force plates for 10 s and then to start walking at his own will, after a “ready?” vocal prompt. GI was performed with the preferred right foot, at natural speed. Both tasks were performed barefoot and after wearing ad-hoc prosthetic shoes (S.C.A.R.P.A. SpA, Asolo, Italy; see Figure 1), which restored the foot original length and were specially designed to provide elastic support during the propulsive phase of walking. For each condition, three trials were collected for SP and ten for GI.

### 2.3 Recordings

Body kinematics were recorded with an eight-camera optoelectronic system (SMART-DX, BTS, Milan, Italy) by placing markers on the heels, malleoli, and heads of the first and fifth metatarsals. A marker was also placed on the stump of the big toe to outline, together with the other markers, the physical limits given by the contour of the feet. Four further markers were placed bilaterally on acromia and anterior-superior iliac spines, ASIS (Figure 1B). In the shod condition, foot markers were located in correspondence to the projections of the same anatomical landmarks on the shoes, except for the malleoli markers which remained in place. Markers were used to calculate the position of the Center of Pressure (CoP) relative to the ankle joint centers in SP and to monitor heel-off events during GI. Two dynamometric force plates (9286AA, KISTLER, Winterthur, Switzerland) allowed to measure the CoP and the Ground Reaction Forces (GRF) for each foot. Wireless probes (FREEEMG 1000, BTS, Milan, Italy) were employed bilaterally to record the surface electromyographic (EMG) activity of Tibialis Anterior (TA), Soleus (Sol), Obliquus Abdominis (OA), Erector Spinae at L2 vertebra (ES-L), Vastus Medialis (VM) and Biceps Femoris (BF). Electrodes were placed according to the Surface Electromyography for the Non-Invasive Assessment of Muscles (SENIAM) guidelines (Hermens et al., 2000). Synchronous data acquisition was accomplished by the SMART-DX workstation; sampling rate being 100 Hz for optoelectronic cameras, 400 Hz for dynamometric signals, and 1000 Hz for EMGs.

### 2.4 Data processing and extracted parameters

For each foot, the CoP pathway and the GRF were filtered with a 4th-order Butterworth lowpass (cut off frequency: 10 Hz) (Santos and Duarte, 2016; Duarte and Freitas, 2010). A weighted mean of the left and right CoP was applied to obtain the whole-body CoP from the signals recorded by the two force plates, as follows (Winter, 1995):

$$C\,o\,P=\frac{C\,o\,P_{R}\,x\,V\,G\,R\,F_{R}+C\,o\,P_{L}\,x\,V\,G\,R\,F_{L}}{V\,G\,R\,F_{R}+V\,G\,R\,F_{L}}$$

Where *CoP*_*R*_ and *CoP*_*L*_ are the CoP pathways under the right and left foot, respectively, and *VGRF*_*R*_ and *VGRF*_*L*_ are the vertical components of right and left GRF, respectively.

To standardize the absolute position of the CoP and allow inter-trial averaging, the coordinates recorded in the laboratory reference system were re-referred to the vertical projection on the ground of the midpoint between the centers of the ankles, which represents the fulcrum in the inverted pendulum body model (cfr. Winter, 1995).

Several indexes were extracted from whole-body CoP data to quantify the postural control of the subject in SP. These indexes were: the total length of the trajectory, the Anteroposterior (AP) and the Mediolateral (ML) position, the peak-to-peak AP and ML range, the average velocity and the Ellipse area (Prieto et al., 1996). The AP and ML positions and the length of the trajectory were also calculated for both *CoP*_*R*_ and *CoP*_*L*_.

Data from EMG recordings in GI were processed as in Farinelli et al. (2021). In brief, after high-pass filtering (50 Hz for all muscles except OA, in which 150 Hz were used to reject cardiac artifacts), traces were full-wave rectified, then time-aligned to the heel-off of the leading foot and averaged across trials. Time 0 was assigned to the beginning of the backward shift of the CoP. The EMGs were integrated with a 25 ms moving average window, the mean level and SD of the trace from 3 to 1 s before the CoP shift was then measured. APA onsets in EMG traces were identified by a custom-made algorithm, which searched for time points where the EMG crossed a threshold defined as mean +2 SD for excitation and mean −2 SD for inhibition and remained above or below that value for at least 50 ms.

## 3 Results

### 3.1 Postural parameters in SP

Analysis of static posturography (Figures 1C, D and Table 1) showed that, in the barefoot condition, the position of the CoP was shifted more anteriorly than the origin (i.e., the midpoint of the two ankle joint centers). The special anatomical situation associated with toe amputation resulted in a reduced AP extension of the base of support. This base of support is further reduced in the ML direction, as the participant placed the two feet at a shorter distance from each other, with respect to the shod condition. However, body weight distribution was not affected by the conditions. In fact, in both cases, the left limb was about 3% more loaded than the right limb and the *CoP*_*L*_ excursion was larger, consequently the whole-body CoP was shifted to the left. Nevertheless, CoP oscillations, in AP and ML directions, were similar in the two conditions.

**TABLE 1:** Postural parameters of the whole-body center of pressure (CoP) and of the CoP for each separate foot (right and left) in SP.

		Barefoot	Shod
Whole body	CoP AP position (mm)	44.65 (40.26 – 50.82)	37.54 (31.83 – 42.18)
	CoP ML position (mm)	−5.77 (−6.61 – −4.23)	−3.22 (−5.49 – 1.58)
	CoP length (mm)	370.50 (357.96 – 412.61)	352.42 (343.26 – 392.34)
	CoP AP range (mm)	17.78 (16.06 – 23.57)	26.09 (20.99 – 26.94)
	CoP ML range (mm)	14.90 (11.16 – 14.94)	12.42 (7.92 – 13.8)
	CoP Total velocity (mm/s)	12.35 (11.93 – 13.75)	11.75 (11.44 – 13.08)
	CoP Ellipse area (mm^2^)	198.51 (170.22 – 252.19)	208.72 (152.73 – 322.31)
	Δ weight right-left foot (%)	−2.88 (−3.3 – −2.04)	−2.72 (−4.6 – 0.78)
Right side	CoP AP position (mm)	44.55 (42.78 – 47.17)	35.19 (33.2 – 40.1)
	CoP ML position (mm)	117.62 (112.27 – 118.78)	136.16 (133.2 – 136.48)
	CoP length (mm)	229.33 (224.79 – 281.92)	210.92 (204.19 – 321.27)
	AJC AP position (mm)	−1.04 (−4.21 – 0.23)	2.36 (−2.49 – 0.71)
	AJC ML position (mm)	112.25 (108.47 – 113.73)	124.48 (121.52 – 126.13)
Left side	CoP AP position (mm)	44.8 (37.84 – 54.26)	39.87 (30.29 – 44.28)
	CoP ML position (mm)	−122.92 (−123.34 – −116.07)	−135.02 (−135.24 – −132.09)
	CoP length (mm)	497.48 (489.96 – 581.57)	492.42 (451.91 – 497.12)
	AJC AP position (mm)	1.04 (−0.23 – 4.21)	2.36 (−0.71 – 2.49)
	AJC ML position (mm)	−112.25 (−113.73 – −108.47)	−124.48 (−126.13 – −121.52)

Data are shown as median (min-max). AP, anteroposterior, positive forward; ML, mediolateral, positive towards the right foot, AJC, ankle joint center.

### 3.2 EMG activities in GI

**FIGURE 2:**
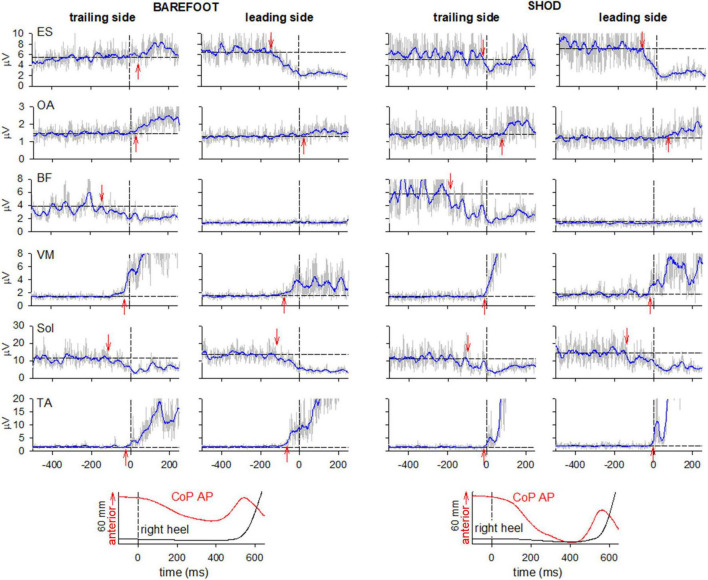
Comparison of raw (gray) and integrated (tc = 25 ms, blue) EMG activity in barefoot (left) and shod (right) condition on the leading and trailing sides. From the top: Erector Spinae at L2 vertebra (ES), Obliquus Abdominis (OA), Biceps Femoris (BF), Vastus Medialis (VM) and prime movers Tibialis Anterior (TA) and Soleus (Sol). Arrows mark the onset of excitation (upward arrow) or inhibition (downward arrow). Black dashed lines at time 0 mark the onset of the backward whole-body CoP shift while the horizontal lines represent the average muscle activity at baseline. The two lowermost plots show the anteroposterior CoP displacement and the vertical displacement of the right heel marker.

In both barefoot and shod conditions (Figure 2), APAs in trunk and leg muscles accompanied the well-known reciprocal pattern of prime movers’ excitation in TA and inhibition in Sol.

When barefoot, ES was excited in the trailing left side while inhibited the leading right side. On the contrary, when shod, ES was bilaterally inhibited. No changes were observed in the excitatory pattern bilaterally expressed in OA and VM in both conditions. Also, BF showed a similar pattern of inhibition in the trailing side and no activity in the contralateral one.

**FIGURE 3:**
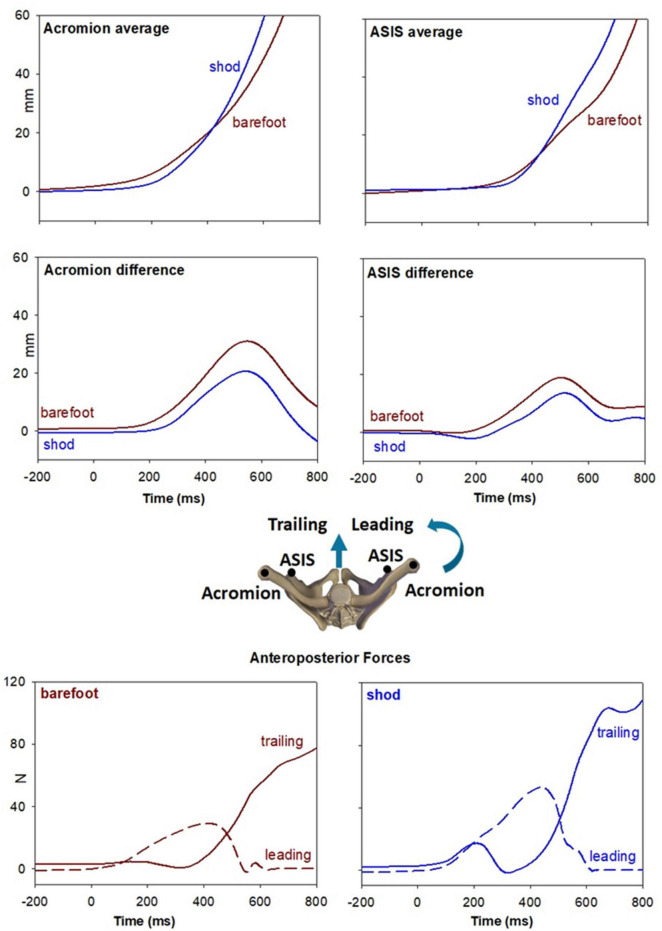
Top panel illustrate the forward projection of scapular girdle and pelvis evaluated by the average AP displacement of acromia and ASISs markers (see inset). Brown traces refer to the barefoot condition while blue traces to the shod. Middle panels show the rotation of the same segments toward the trailing side, evaluated by right minus left difference in AP displacement of the same markers. Bottom panels show the AP GRFs under each foot in the two conditions.

Figure 2 also reports, as last traces, the vertical coordinate of the right heel marker and the anterior-posterior displacement of the whole-body CoP. Despite the similar latency between CoP onset and heel-off, it is apparent that in shod condition the CoP displacement was much larger and faster than when barefoot. Together with the changes in ES activity (from bilateral inhibition to excitation on the left and inhibition on the right), this behavior highlights a different GI strategy in the two conditions. This aspect was further investigated by extracting the kinematics of acromion and ASIS as well as the GRF under each foot (Figure 3). GI is accompanied by a forward projection of the scapular girdle and pelvis, together with a rotation toward the trailing side. Such movements are apparent from the average AP displacement of acromia and ASISs (top panels) and from the difference between markers of the two sides (middle panels), respectively. Note however, that the rotation was larger and earlier when barefoot while the forward projection lately prevailed in shod. This is also confirmed by the GRF traces: when barefoot (left bottom panel) a forward force developed under the leading foot but not under the trailing one, while in shod (right bottom panel) forward GRFs simultaneously started at the two feet.

## 4 Discussion

One of the aims of the present study was to quantify the residual deficits in static and dynamic balance when barefoot; this could be derived by comparing static posturography data with current literature. Reference values may be obtained from Prieto et al. (1996) who studied both young and older healthy individuals (21–35 years, 26.4 ± 4.9 SD *vs.* 66–70 years, 68 ± 1.3 SD), considering the last 20 s of a total 30 s recording period and applying a cut-off frequency of 5 Hz. Thus, we recalculated the CoP AP & ML range as well as Total velocity and Ellipse area according to Prieto’s methods. When barefoot, our participant showed a CoP ML range and Ellipse area which were within a ± 15% range with respect to those found in healthy young individuals. Instead, CoP AP range and Total velocity were about 50% greater, reaching values of older subjects. It is interesting to note that the main impairment regarded the AP direction, a result seemingly linked to the AP reduction in the base of support.

Another source of information comes from the average position of the CoP relative to the midpoint of ankle joint centers, a quite novel measurement approach. In fact, it is well known that healthy individuals place their CoP near such origin point. Our participant, instead, placed the CoP much more anteriorly, close to the geometrical center of the base of support. This compensation strategy grants a larger margin of stability, although the energetic cost of a stronger activity in the soleus muscles. Interestingly enough, when prosthetic shoes restored the normal foot length, the CoP moved backward, closer to the ankle’s midpoint, but the stability did not overall improve.

Apart from the mechanic consequences, toes amputation clearly affected the sensory inflow, which is known to play an important role in both controlling weight distribution in the static posture and in governing the postural reflexes in walking (Wright et al., 2012; Kavounoudias et al., 1998; Fallon et al., 2005). Actually, the situation may be reconducted in the framework of reduced afferent inflow which characterizes several diseases, like neuropathies and traumatic nerve lesions, whose effects on postural stability and gait has been extensively studied (Felicetti et al., 2021). This in agreement with the observation that the stability in AP direction of our 52 years old amputee was comparable to that of quite older healthy individuals. It remains to be ascertained whether the CoP shifted forward in the barefoot condition in search of a higher afferent inflow from the residual forefoot and/or for approaching the center of the base of support to grant a greater margin of stability.

Finally, an important outcome of the present study regards the change in the gait initiation strategy between barefoot and shod conditions. Indeed, the analysis of back muscles EMG together with shoulder and pelvis kinematics and GRFs demonstrated that in barefoot condition GI is mainly accomplished by forward projecting the leading body side while fixing the contralateral trunk side (excitatory APA in ES) which then became a pivot. When shod instead, bilateral ES inhibition promoted a symmetric forward “dive”. Considering that GI was slower when barefoot, the “turning” approach may represent, as the forward CoP shift in SP, a search for a safer strategy in accomplishing the motor task. Conversely, the self-confidence linked to the prosthetic shoes would promote the faster “diving” approach. It is worth to note that the “turning” strategy is commonly used by untrained healthy young individuals (see Farinelli et al., 2021), while we observed that highly physical active people mainly adopted the “diving” strategy (manuscript in preparation). These observations are also in agreement with the finding that about 2 months after the onset of independent walking, well before stabilizing the “adult” pendulum mechanism, toddlers display different strategies for body progression (Bisi and Stagni, 2015): besides the more common “stepper” strategy, also described as the “puppet’s march”, some toddlers twisted the trunk, similarly to our “turning” approach, while others adopted a controlled forward fall, like in our “diving” approach. This indicates that different patterns are available and may be exploited according to the postural requirements.

In conclusion, the outcome of the present case report is two-fold: on one side it allowed us to describe the postural control in a rare case of bilateral amputation of all the toes, on the other side it provided the novel evidence that one and the same individual may adopt different strategies in accomplishing a natural and only seemingly stereotyped task like gait initiation.

## Data Availability

The raw data supporting the conclusions of this article will be made available by the authors, without undue reservation.
